# Long non-coding RNA *C2dat1* regulates CaMKII*δ* expression to promote neuronal survival through the NF-*κ*B signaling pathway following cerebral ischemia

**DOI:** 10.1038/cddis.2016.57

**Published:** 2016-03-31

**Authors:** Q Xu, F Deng, Z Xing, Z Wu, B Cen, S Xu, Z Zhao, R Nepomuceno, M I H Bhuiyan, D Sun, Q J Wang, A Ji

**Affiliations:** 1 Department of Pharmacology and Chemical Biology, University of Pittsburgh School of Medicine, Pittsburgh, PA, USA; 2 Department of Pharmacy, Zhujiang Hospital, Southern Medical University, Guangzhou, China; 3 Department of Cell Biology, School of Basic Medical Sciences, Southern Medical University, Guangzhou, China; 4 Department of Neurology, University of Pittsburgh, Pittsburgh, PA, USA

## Abstract

Increasing evidence has demonstrated a significant role of long non-coding RNAs (lncRNAs) in diverse biological processes. However, their functions in cerebral ischemia remain largely unknown. Through an lncRNA array analysis in a rat model of focal cerebral ischemia/reperfusion (I/R), we have identified *CAMK2D*-associated transcript 1 (*C2dat1*) as a novel I/R-induced lncRNA that regulated the expression of CaMKII*δ* in murine models of focal cerebral ischemia. *C2dat1* mRNA was upregulated in a time-dependent manner in mouse cortical penumbra after focal ischemic brain injury, which was accompanied by increased expression of CaMKII*δ* at transcript and protein levels. The expression patterns of *C2dat1* and *CAMK2D* were confirmed in mouse Neuro-2a cells in response to *in vitro* ischemia (oxygen-glucose deprivation/reoxygenation, OGD/R). Knockdown of *C2dat1* resulted in a significant blockade of CaMKII*δ* expression, and potentiated OGD/R-induced cell death. Mechanistically, reduced CaMKII*δ* expression upon silencing *C2dat1* inhibited OGD/R-induced activation of the NF-*κ*B signaling pathway. Further analysis showed that the downregulation of IKK*α* and IKK*β* expression and phosphorylation, and subsequent inhibition of I*κ*B*α* degradation accounted for the inhibition of the NF-*κ*B signaling activity caused by silencing *C2dat1*. In summary, we discovered a novel I/R-induced lncRNA *C2dat1* that modulates the expression of CaMKII*δ* to impact neuronal survival, and may be a potential target for therapeutic intervention of ischemic brain injury.

Stroke is caused by a sudden disruption of blood flow to the brain, and approximately 85% of all strokes are due to cerebral ischemia resulting from embolic or thrombotic occlusion of a major cerebral artery. Despite the efforts on developing the pharmacological and surgical treatments of the disease, tissue plasminogen activator (tPA) is the only effective therapy at present. A better understanding of the pathological process and the discovery of new targets and therapies will significantly advance the field.

Non-coding RNAs (ncRNAs) comprise a large portion of the transcribed genome and regulate many important biological processes. Increasing evidence has demonstrated an important role of ncRNAs, such as microRNAs (miRNAs), as critical regulators of stroke-associated biological processes.^1, 2^ However, the expression and function of long ncRNAs (lncRNAs) in stroke and neuroprotection remain largely unknown.^3^ LncRNAs are ncRNAs >200 nucleotides long and do not encode proteins. Although only a handful of lncRNAs have been fully characterized, it is clear that they are involved in diverse biological processes. It has been shown that the expression of lncRNA is developmentally regulated and can be tissue- and cell-type specific. In the central nervous system (CNS) in particular, lncRNAs have been demonstrated to be one of the most abundant classes of ncRNAs and their expression is spatially restricted and temporally regulated, indicating an important role of lncRNAs in brain development.^4, 5, 6, 7^ Growing evidence has also demonstrated the functional significance of lncRNAs in neuronal differentiation, maintenance and plasticity.^8, 9^

Calcium/calmodulin-dependent kinase II (CaMKII) is a family of serine/threonine kinases consisting of four isoforms (CaMKII*α*, *β*, *γ* and *δ*) encoded by different genes and display distinct and overlapping expression patterns.^10^ CaMKII is one of the most abundant protein kinases in the brain,^11^ CaMKII*α* in particular makes up to >1% of total protein in certain areas of the brain, reflecting a key role of these proteins in neural transduction, synaptic plasticity and brain synapse development.^11, 12^ CaMKII are complex and multifunctional protein kinases with broad substrate specificity, and the holoenzyme is a homo- or heteromultimer that is assembled by 8–12 isoforms. CaMKII is activated by Ca^2+^ influx through the activation of the glutamate receptors in neurons. Ischemic conditions can trigger massive release of glutamate leading to ‘glutamate excitotoxicity' involving Ca^2+^ overload, and subsequent neuronal cell death, which accounts for much of the neuronal damage after cerebral ischemia.^12, 13^ It has been shown that after focal ischemia CaMKII is quickly activated and then inactivated in a time- and location-dependent manner, and the extent of neuronal damage correlates with the degree of loss of CaMKII activity.^14, 15, 16^ Meanwhile, CaMKII*α* knock-out mice display increased infarct volume than that of wild-type litter mates.^17^ The studies from the Hudmon group have also showed that prolonged pharmacological inhibition of CaMKII promotes neuronal death by increasing neuronal vulnerability to glutamate,^18, 19^ which is in contrast to short-term inhibition of CaMKII that protects neurons from excitotoxic insult.^19^ Other studies have also shown that acute inhibition of CaMKII before excitotoxic insults, such as glutamate challenge or hypoxia/hypoglycemia treatment, was neuroprotective *in vitro* and *in vivo*.^19, 20, 21, 22, 23, 24^ The mechanisms underlying the paradoxical effects of CaMKII inhibition/depletion are not well understood. Moreover, despite extensive studies on the abundantly expressed CaMKII*α* or CaMKII holoenzyme, the function of CaMKII*δ*, one of the most ubiquitous CaMKII isoforms expressed both in neuronal and non-neuronal cells, remains largely unknown in the CNS.^10, 25^

In this study, we report the discovery of a novel *CAMK2D*-associated lncRNA, *CAMK2D*-associated transcript 1 (*C2dat1*). This lncRNA was upregulated in murine ischemia/reperfusion (I/R) models and in mouse neuronal cells upon *in vitro* ischemia oxygen-glucose deprivation/reoxygenation (OGD/R). *C2dat1* regulated the expression of *CAMK2D*/CaMKII*δ* expression in response to OGD/R. *C2dat1*-induced CaMKII*δ* expression promoted neuronal survival by activating the NF-*κ*B signaling pathway. The neuroprotective role of *C2dat1* may be exploited for therapeutic intervention of I/R-induced neuronal injury.

## Results

### *C2dat1* was a novel *CAMK2D*-associated lncRNA induced by I/R

**Figure 1:**
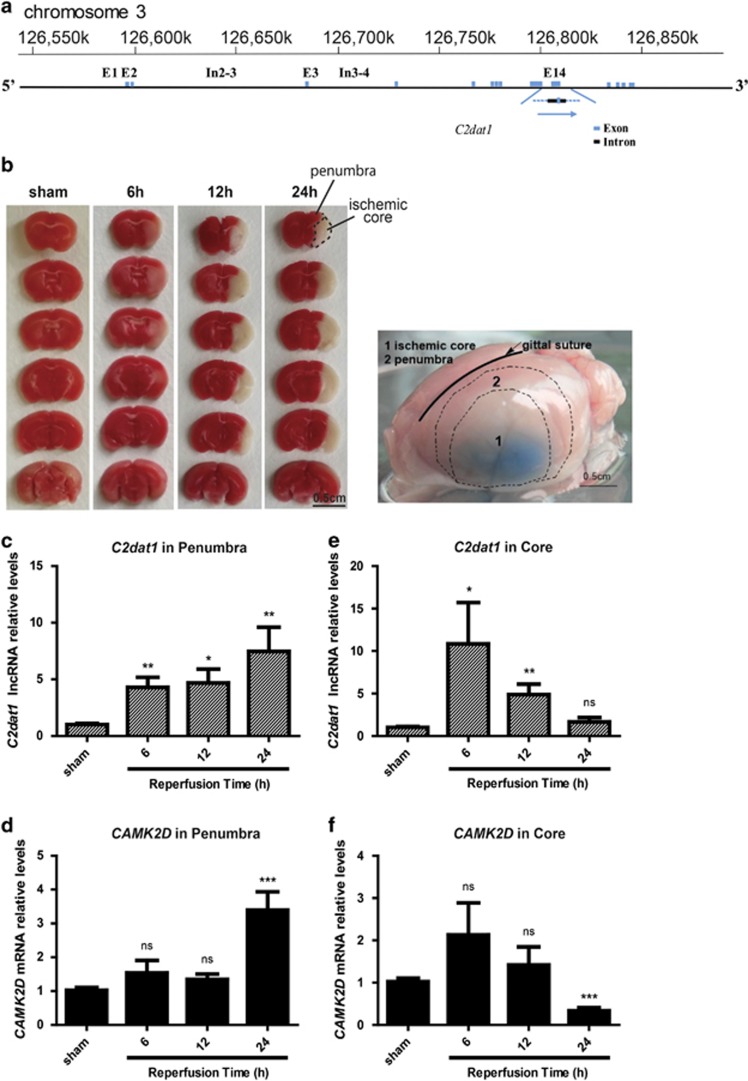
I/R-induced *C2dat1* and *CAMK2D* transcription in mouse model of I/R. (**a**) Genomic locus of *C2dat1*. *C2dat1* contains overlapping sequences from introns 13 to 15 and exon 14 of *CAMK2D* in sense direction. (**b**) Focal ischemia induced by MCAO resulted in progressive brain damage that was visible at 12 and 24 h. Representative coronal brain sections stained with TNN are shown. Right, the image showing the anatomical location of the ischemic core and penumbra in the cortex. 1, ischemic cortex; 2, ischemic penumbra. (**c-f**) Transcript levels of *C2dat1* and *CAMK2D* in ischemic cortex and the surrounding penumbra in sham and ischemic tissues. *C2dat1* (**c** and **e**) and *CAMK2D* (**d** and **f**) were measured by real-time RT-qPCR. Data are the mean±S.E.M. of three independent experiments. *P<0.05; **P<0.01; ***P<0.001; NS, not significant

After focal cerebral ischemia, massive cell death occurs in the ischemia core in an irreversible manner; however, in the surrounding penumbra the injury was less severe and may be reversible upon therapeutic intervention. We examined the lncRNA expression profiles of the rat brains subjected to transient focal ischemia via middle cerebral artery occlusion (MCAO) using microarray.^26, 27^ MCAO induced time-dependent irreversible cell death in the ischemic core in 6–24 h after transient focal ischemia in rats and mice. We specifically determined changes of lncRNA expression profiles in the penumbra areas using microarray (Wu *et al.*, unpublished results). In all, 1187 lncRNAs (360 up- and 827 downregulated) and 1894 mRNAs (1081 up- and 813 down-regulated) were found to be differentially expressed (⩾2-fold change, *P*⩽0.05). Eighty-one lncRNAs showed >5-fold changes (46 up- and 35 down-regulated) (Wu *et al.*, unpublished results). A panel of differentially expressed lncRNAs was then selected for validation in mouse Neuro-2a (N2a) cells subjected to *in vitro* ischemia (OGD/R) (see Supplementary Table S2 for selected lncRNAs). The lncRNA detected by AK153573 was among a few validated lncRNAs that showed consistent changes in cellular and rat/mouse models of I/R, which we later named it as CaMKII*δ*-associated transcript I (*C2dat1*). The known sequence of *C2dat1* overlaps in part with introns 13–15 and exon 14 of *CAMK2D* on mouse chromosome 3, implying that *C2dat1* is a *CAMK2D*-associated lncRNA that may regulate *CAMK2D* gene expression (Figure 1a). Taken together, focal cerebral ischemia caused genome-wide alternations of lncRNA expression in ischemic penumbra, and *C2dat1* as a novel I/R-induced lncRNA could have a role in the pathophysiology of ischemia.

### Focal ischemia caused the upregulation of *C2dat1* and *CAMK2D* in mice

The expression of *C2dat1* and *CAMK2D* were assessed in a mouse model of I/R. Focal cerebral ischemia was induced in mice by MCAO for 1 h, followed by 24-h reperfusion.^26, 27^ Brain tissues from the ischemic core and surrounding penumbra were obtained at different time points (6, 12 and 24 h) and subjected to RNA extraction and real-time RT-qPCR. Focal ischemia caused progressive cell death in the ischemic core and was visible at 12 and 24 h (Figure 1b). In the ischemic core, both *C2dat1* and *CAMK2D* transcripts were abruptly upregulated at 6 h, and then gradually downregulated to basal levels at 24 h, in line with the massive neuronal death in this area (Figures 1e and f). In contrast, penumbra exhibited a time-dependent upregulation of *C2dat1* and *CAMK2D* with an over sevenfold increase for *C2dat1* and a nearly fourfold increase for *CAMK2D* at 24 h after ischemia (Figures 1c and d). In summary, focal ischemia induced a parallel increase in *C2dat1* and *CAMK2D* mRNA in mouse model of I/R with differential patterns in ischemic core and penumbra regions.

### Focal ischemia caused the upregulation of CaMKII*δ* in neurons at the peri-infarct region and in the primary cortical neuron cultures

**Figure 2:**
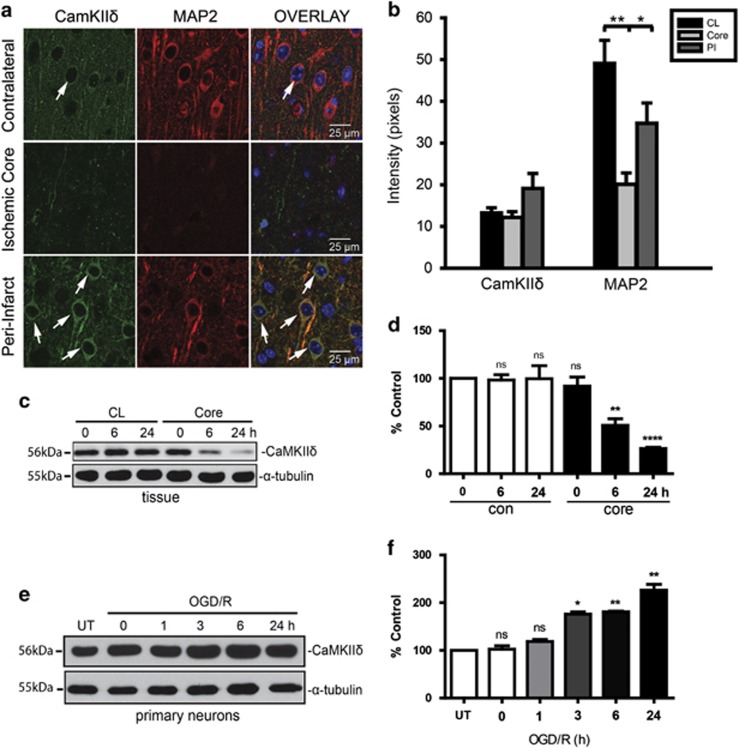
Focal ischemia caused the upregulation of CaMKII*δ* in neurons at the PI region and in the primary cortical neuron cultures. (**a**) Fluorescence immunostaining of CaMKII*δ* (green) and the neuronal marker MAP-2 (red) in ischemic core and PI (penumbra) regions of the cortex after MCAO. Representative immunofluorescence images are shown. Arrow depicts neurons with increased expression of CaMKII*δ*. (**b**) CaMKII*δ* and MAP-2 immunoreactivity were quantified by measuring the fluorescence intensity of images in **a**. At least, three independent areas were quantified from each mouse brain slice. Core, ischemic core. Values are expressed as mean±S.E.M. (*n*=5), *P<0.05; **P<0.01. (**c**) Expression of CaMKII*δ* at 0, 6 and 24 h post ischemia in the CL and ischemic core (Core). Representative images from one of three independent experiments are shown. (**d**) Quantitative measurement of band intensity in **c** by densitometry analysis. Data are the mean±S.E.M. of three independent experiments. **P<0.01; ****P<0.0001; ns, not significant. (**e**) Expression of CaMKII*δ* in untreated and OGD/R-treated primary neurons at different time points. Representative images from one of three independent experiments are shown. (**f**) Quantitative measurement of band intensity in **e** by densitometry analysis. Data are the mean±S.E.M. of three independent experiments. *P<0.05; **P<0.01

The expression of CaMKII*δ* was examined in the ischemic core and the peri-lesion regions (penumbra) of mouse cerebral cortex after I/R by immunofluorescence staining. As shown in Figure 2a, CaMKII*δ* was upregulated and colocalized with the neuronal marker microtubule-associated protein-2 (Map2) in neurons of peri-infarct (PI) region after I/R. Quantitative measurement of the fluorescence intensity of CaMKII*δ* in the contralateral (CL) side, the ischemic core (Core) and PI region indicated that CaMKII*δ* was upregulated in the PI region (Figure 2b). In contrast, CaMKII*δ* was significantly downregulated in ischemic core after I/R (Figures 2a and c). Western blot analysis of the tissues from the ischemic region (ischemic core) and the CL side showed rapid and progressive decrease of CaMKII*δ* expression in the ischemic core in response to I/R, whereas its levels in the CL side remained unchanged at all time points (Figures 2c and d). Next, the levels of CaMKII*δ* were evaluated in the primary cortical neuronal cultures subjected to OGD/R. Consistent with the previous findings, we observed about twofold time-dependent upregulation of CaMKII*δ* in the primary neurons in response to OGD/R (Figures 2e and f). These data implied a potential role of CaMKII*δ* in ischemic-associated biological processes in mice.

### *C2dat1* and *CAMK2D*/CaMKII*δ* were upregulated in response to OGD/R in mouse neuronal cells

**Figure 3:**
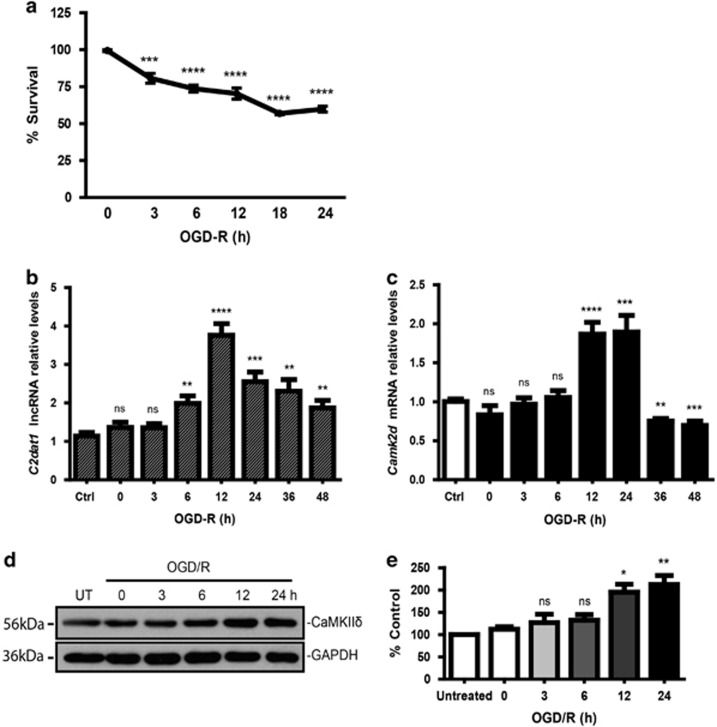
OGD/R-induced *C2dat1* positively correlated CaMKII*δ* expression in mouse neuronal cells. (**a**) OGD/R-induced cell death in N2a cells. Cell survival was measured by CCK-8 assay in N2a cells before and 3-24 h after OGD/R. (**b** and **c**) *C2dat1* and *CAMK2D* were upregulated in response to OGD/R. *C2dat1* (**b**) and *CAMK2D* (**c**) transcripts were measured by real-time RT-qPCR. (**d**) Western blot analysis showed that CaMKII*δ* expression was elevated in response to OGD/R in N2a cells. Representative images from one of three independent experiments are shown. (**e**) Quantitative analysis of the results from **d**. The above data in ‘**b**, **c** and **e**' are the mean±S.E.M. of at least three independent experiments with triplicate determinations at each point.*P<0.05; **P<0.01; ***P<0.001; ****P<0.0001; ns, not significant

Using mouse neuroblastoma (N2a) cells, we further evaluated the expression of *C2dat1* and *CAMK2D*/CaMKII*δ* in response to *in vitro* ischemia. It has been reported that N2a cells express glutamate receptors^28^ and exhibit glutamate receptor-mediated Ca^2+^ overload and excitotoxicity.^29, 30^ Thus, excitotoxicity-induced cell death may have an important role in OGD/R-induced cell death in N2a cells. We first examined the effect of OGD/R on N2a cell survival. As shown in Figure 3a, OGD/R time-dependently induced cell death in N2a cells, which plateaued at about 40% cell death at 24 h post OGD. Second, cells were collected at different time points of reoxygenation and the levels of *C2dat1* and *CAMK2D* transcripts were measured by real-time RT-PCR. OGD/R caused the upregulation of *C2dat1* over time, which peaked at about 12 h and then declined within 48 h (Figure 3b). Accordingly, *CAMK2D* was also upregulated in response to OGD/R, which peaked at 12 and 24 h, and then returned to baseline at about 36 and 48 h (Figure 3c). A persistent and time-dependent upregulation of CaMKII*δ* was also detected at protein level (~ 2-fold) (Figures 3d and e). Thus, OGD/R induced the upregulation of *C2dat1* and *CAMK2D/CaMKIIδ* in mouse neuronal cells.

### Knockdown of *C2dat1* blocked the transcription and protein expression of CaMKII*δ* and enhanced neuronal death

**Figure 4:**
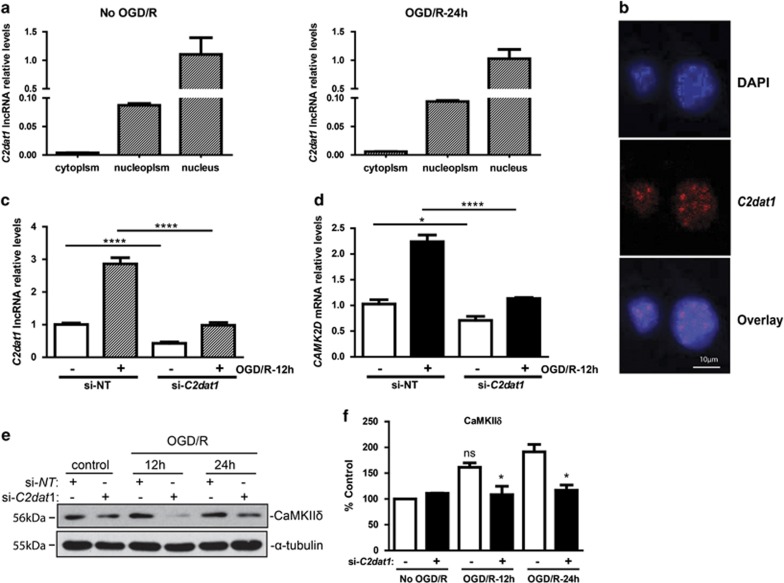
*C2dat1* was primarily localized in the nucleus of N2a cells and knockdown of *C2dat1* caused the downregulation of *CAMK2D* mRNA and protein. (**a**) Majority of *C2dat1* was in the nuclear fraction. N2a cells were fractionated to cytosol, nucleoplasm, and nucleus fractions. Levels of *C2dat1* were quantified by real-time RT-PCR at basal state. (**b**) *C2dat1* was detected in the nucleus of N2a cells post 12-h OGD/R by RNA FISH. (**c** and **d**) Knockdown of *C2dat1* abolished OGD/R-induced *CAMK2D* expression. N2a cells were transfected with an si-*C2dat1* and a non-targeting siRNA (si-NT) at 60 nM. Two days after transfection, cells were subjected to OGD for 3 h and then 12-h reoxygenation. The transcript levels of *C2dat1* (**c**) and *CAMK2D* (**d**) were examined by real-time RT-qPCR. (**e**) Knockdown of *C2dat1* blocked the upregulation of CaMKII*δ* by OGD/R. N2a cells were similarly transfected with si-*C2dat1* and si-NT. Following OGD/R treatment, the levels of CaMKII*δ* was examined by western blot analysis. The *α*-tubulin was blotted as loading control. The experiments were repeated at least three times and data from a representative experiment are shown. (**f**) Quantitative analysis of the results from **e**. The above data in ‘**a**, **c**, **d** and **f**' are the mean±S.E.M. of three independent experiments performed in triplicate.*P<0.05; ****P<0.0001; ns, not significant

The intracellular localization of lncRNA is critical to its biological function. To evaluate the subcellular localization of *C2dat1*, N2a cells were fractionated into cytosolic and nuclear fractions. Levels of *C2dat1* were measured before and 24 h after OGD/R by real-time RT-PCR. Our data showed that *C2dat1* was predominantly expressed in the nucleus of N2a cells (Figure 4a). This finding was confirmed by RNA fluorescence *in situ* hybridization (FISH) analysis. As shown in Figure 4b, *C2dat1* was predominantly located in the nucleus of N2a cells at 12 h post OGD/R and at basal state as measured by RNA FISH. Thus, C2dat1 was a nucleus-localized lncRNA.

The overlapping sequences suggest that *C2dat1* may regulate *CAMK2D*/CaMKII*δ* expression. To test this hypothesis, a *C2dat1* siRNA was designed to target the region of the lncRNA outside of the overlapped sequences with *CAMK2D* exon 14. N2a cells were transfected with the *C2dat1*-targeting siRNA (si-*C2dat1*), which resulted in a significant knockdown of *C2dat1* transcript at basal state (data not shown) and 12 h post OGD/R (Figure 4c). Accordingly, knockdown of *C2dat1* significantly blocked OGD/R-induced *CAMK2D* expression (Figure 4d). At protein level, knockdown of *C2dat1* significantly decreased CaMKII*δ* expression in N2a cells before and after OGD/R (Figures 4e and f). Note that, as there are no overlapping sequences between *C2dat1* and other CaMKII isoforms (CaMKII*α*, *β*, *γ*), it is unlikely that the expression of these CaMKII isoforms is regulated by *C2dat1*. In addition, it is known that each *CAMK2* gene gives rise to multiple isotypes by alternative splicing, and at least 10 *CAMK2D*/CaMKII*δ* isotypes have been described.^31^ Sequence analysis indicates that the exon 14 of *CAMK2D* that overlaps in part with *C2dat1* encodes the variable domain of CaMKII*δ*, and this regions of *CAMK2D*_1/A_ and *CAMK2D*_4_ aligns with that of *C2dat1*, suggesting that *C2dat1* may selectively target CaMKII*δ*_1/A_ and CaMKII*δ*_4_. Using primers that selectively detect the transcripts of *CAMK2D*_1/4_, *CAMK2D*_2/3_ and *CAMK2D*_5/9_, we then examined the mRNA levels of *CAMK2D* isotypes in response to OGD/R with or without knockdown of *C2dat1*. Knockdown of *C2dat1* abolished IR-induced expression of *CAMK2D*_1/4_, partially inhibited that of *CAMK2D*_5/9_, but had no effect on *CAMK2D*_2/3_, implying that *C2dat1* could regulate the expression of *CAMK2D*_1/4_ selectively (Supplementary Figure S1). Taken together, *C2dat1* regulated the expression of *CAMK2D*/CaMKII*δ* in response to OGD/R in mouse neuronal cells.

### *C2dat1* promoted neuronal survival through the activation of the NF-*κ*B signaling pathway

**Figure 5:**
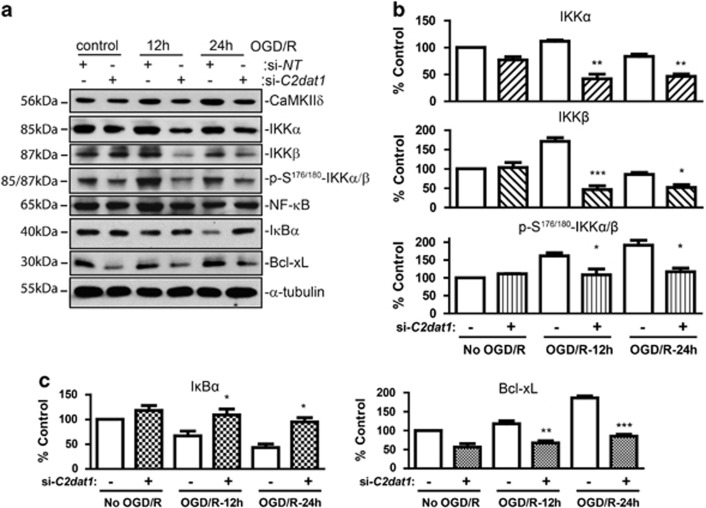
OGD/R-induced *C2dat1* activated the NF-*κ*B signaling pathway. (**a**) Effects of *C2dat1* knockdown on CaMKII*δ* expression and the NF-kB pathway. N2a cells were transfected with si-*C2dat1* and si-NT. The transfected cells were subjected to OGD/R, followed by immunoblotting for components of the NF-kB signaling pathway. Representative images from one of at least three independent experiments are shown. (**b** and **c**) Quantification of the native and phosphorylated IKK*α*/*β* (**b**), I*κ*B*α* and Bcl-xL levels (**c**). The western blots from at least three independent experiments were evaluated by densitometry analysis and the mean±S.E.M. from these data were calculated and plotted.*P<0.05; **P<0.01; ***P<0.001

To gain insights into the signaling events downstream of *C2dat1*, we examined several potential downstream signaling pathways involved in I/R-induced neuronal cell death including the NF-*κ*B, Akt, ERK and p38 signaling pathways (Supplementary Figure S2), and the NF-*κ*B signal pathway was consistently altered upon knocking down of *C2dat1*. The NF-*κ*B pathway is one of the main pro-inflammatory signaling pathways that can be activated in response to a variety of stimuli. I/R induces the activation of NF-*κ*B, which has an important role in the inflammatory responses associated with I/R. The pro-inflammatory activities induced by NF-*κ*B often lead to neuronal cell death, however, pro-survival effects of NF-*κ*B has also been reported. The dual role of the NF-*κ*B signal pathway appears to be stimulus- and cell/tissue-type dependent. As shown in Figures 5a and c, in the neuronal N2a cells, the NF-*κ*B signal pathway was activated at 12 and 24 h post OGD, evident by the increased phosphorylation of IKK*α* and IKK*β* measured by the p-S^176/180^-IKK*α*/*β* antibody, followed by decreased I*κ*B*α* protein expression. Knockdown of *C2dat1* inhibited CaMKII*δ* expression, blocked the phosphorylation of IKK*α* and IKK*β* at S^176/180^ and downregulated IKK*α* and IKK*β* protein expression (Figures 5a and b). This resulted in the nearly complete blockade of I*κ*B*α* degradation (Figures 5a and c) and inhibition of the NF-*κ*B signaling activity. Accordingly, Bcl-xL, a NF-*κ*B target gene, was downregulated before and after OGD/R as a result of *C2dat1* knockdown and inhibition of the NF-kB activity (Figures 5a and c).

**Figure 6:**
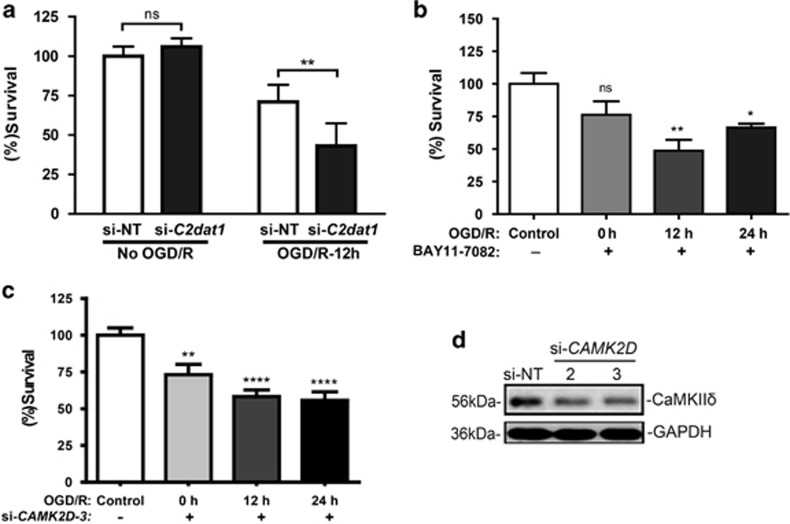
*C2dat1* promoted neuronal cell survival in response to OGD/R. (**a**) Knockdown of *C2dat1* promoted OGD/R-induced cell death. Cell viability was measured by CCK-8 assay before and 12 h after OGD. (**b**) Inhibition of IKK by BAY11-7082 enhanced cell death induced by OGD/R in N2a cells. BAY11-7082 (1 *μ*M) was added at the beginning of reoxygenation right after OGD for a total of 6 h. Cell viability was measured by CCK-8 assay. (**c**) Knockdown of *CAMK2D* potentiated OGD/R-induced cell death. N2a cells were transfected with a *CAMK2D* siRNA (si-*CAMK2D-3*), followed by OGD/R treatment for 0, 12 and 24 h. Cell viability was measured by CCK-8 assay. (**d**) *CAMK2D* siRNAs (si-CAMK2D-2 and -3) caused significantly knockdown of *CAMK2D*/CaMKII*δ*. Cells were transfected for 48 h using 50 nM siRNAs, followed by immunoblotting for CaMKII*δ* and *α*-tubulin. The data are the mean±S.E.M. of at least three independent experiments performed in triplicate.*P<0.05; **P<0.01; ****P<0.0001; ns, not significant

**Figure 7:**
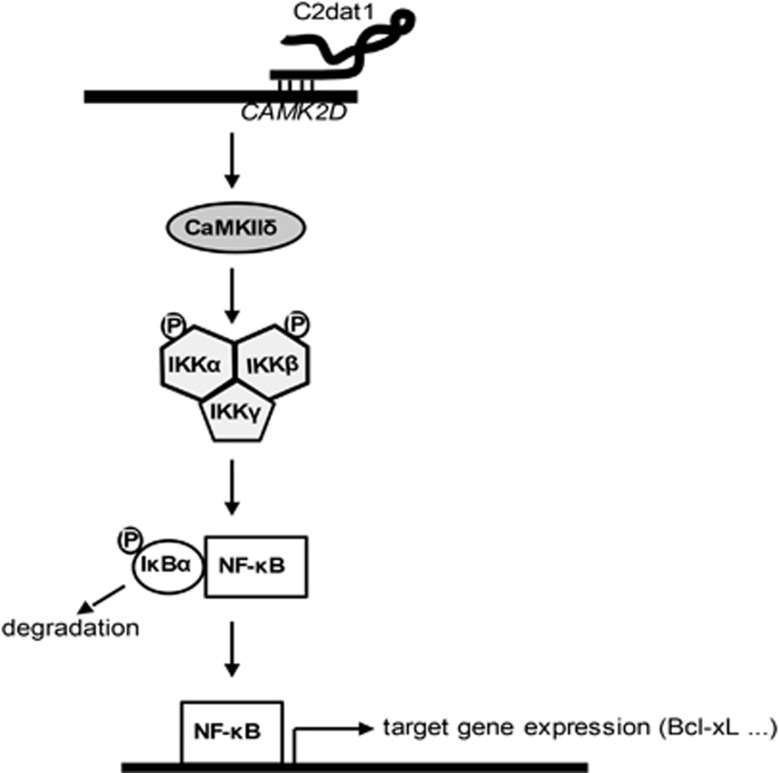
A diagram depicting the signaling mechanisms of *C2dat1*. I/R-induced *C2dat1* upregulates the expression of *CAMK2D* at the transcriptional level, possibly through direct binding to the *CAMK2D* gene via the overlapping sequence. The upregulation of *CAMK2D* led to an increased CaMKII*δ* protein expression and enhanced activity (I/R is known to activate CaMKII). The activated CaMKII*δ* stimulates IKK*α*/*β* protein expression and their phosphorylation at S^176/180^, which resulted in I*κ*B*α* degradation and subsequent NF-*κ*B translocation to the nucleus, where it induces the transcription of multiple genes including Bcl-xL to promote cell survival

The functional impact of *C2dat1* induction on neuronal survival was determined by knocking down this lncRNA using RNAi. As shown in Figure 6a and Supplementary Figure S3A, knockdown of *C2dat1* exacerbated OGD/R-induced neuronal cell death at 12 h post OGD, indicating that the ischemia-induced *C2dat1*/CaMKII*δ*/NF-*κ*B pathway was neuroprotective in N2a cells. Moreover, inhibition of the NF-*κ*B signaling activity by an IKK inhibitor BAY11-7082 resulted in time-dependent cell death in response to OGD/R, implying that activation of the NF-*κ*B signal pathway promotes the survival of N2a cells (Figure 6b and Supplementary Figure S3B). Similarly, knockdown of CaMKII*δ* potentiated cell death induced by OGD/R in a time-dependent manner (Figures 6c and d). The signaling pathway through which *C2da1* regulates neuronal survival in response to ischemia is depicted in Figure 7. Taken together, our data implied that the NF-*κ*B signal pathway was a major target of *C2dat1*-regulated CaMKII*δ* in response to OGD/R in mouse neuronal cells and the activation of NF-*κ*B promoted neuronal survival.

## Discussion

### *C2dat1* in regulation of CaMKII*δ*

Stroke as one of the leading cause of death and adult disability worldwide has incurred significant family and society burdens.^32^ Substantial efforts have been devoted to obtain more details of its cellular and molecular pathophysiology, yet, much remains to be elucidated. LncRNAs, as one of the most abundantly expressed ncRNAs, may have critical roles in stroke-related biological processes. In this study, we report the discovery of *C2dat1*, the first *CAMK2D*-associated lncRNA, induced by I/R in murine focal cerebral ischemic models, which may be neuroprotective during ischemia-induced neuronal injury. There have not been any reports of CaMKII-associated lncRNAs, and *C2dat1* represents the first-ever CaMKII regulatory lncRNA identified thus far. *C2dat1* was identified through an lncRNA array analysis of rat brain tissues subjected to transient focal ischemia. It contains overlapping nucleotide sequences with introns 13–15 and exon 14 of *CAMK2D*. *C2dat1* upregulated the expression of CaMKII*δ* in murine models of I/R and *in vitro* ischemia. This regulation is likely isotype specific as there are no overlapping sequences between *C2dat1* and other CaMKII isoforms (CaMKII*α*, *β*, *γ*). In light of the abundancy and significance of CaMKII in the CNS, the finding of an lncRNA that selectively targets CaMKII*δ* to modulate neuronal survival will have profound biological and therapeutic implications.

There are five different categories of lncRNAs classified based on their positions relative to protein coding genes, including sense, antisense, bidirectional, intronic and intergenic. *C2dat1* is a sense lncRNA with complimentary sequences with the exons and introns of *CAMK2D*. LncRNAs regulate transcription through multiple mechanisms, such as recruiting epigenetic complexes, directly modulating the transcriptional process as decoys, coregulators or interfere with RNA polymerase II activity.^33^ It may also act in post-transcriptional control by regulating mRNA processing and stability.^33^ Our data indicate that *C2dat1* was upregulated in a transient time-dependent manner that peaked at 12 h, which correlated to the gradual elevation of CaMKII*δ* protein in N2a cells subjected to OGD/R and in mouse penumbra under I/R. This kinetics of regulation implies a possible direct transcriptional control via targeting the *CAMK2D* gene. This can be further supported by the nuclear localization of *C2dat1* as determined by RNA FISH. Although it remains to be determined how *C2dat1* regulates *CAMK2D* expression (outside the scope of the current article), we will direct future efforts to further elucidate the molecular basis underlying the regulation of *CAMK2D* by *C2dat1* at the transcriptional level.

### Roles of CaMKII*δ* in neuronal survival

CaMKII*δ* is one of the most ubiquitously expressed CaMKII isoforms in both neuronal and non-neuronal cells and a common component of the CaMKII holoenzyme. Its expression and function in the CNS are not well defined despite numerous studies on CaMKII and CaMKII*α*. In our study, analysis of CaMKII*δ* expression by Western blotting and immunostaining has led to the detection of abundant CaMKII*δ* in N2a cells, primary neuronal cultures and mouse brain sections, implying that CaMKII*δ* may be functionally important to neurons in the brain. I/R and OGD/R-induced gradual and persistent upregulation of CaMKII*δ* at transcript and protein levels, which correlated to the increase of *C2dat1*. Knockdown of *C2dat1* blocked the upregulation of CaMKII*δ*, indicating that *C2dat1* controls the expression of CaMKII*δ* at the transcriptional level. As reduced CaMKII*δ* by silencing *C2dat1* potentiated OGD/R-induced neuronal cell death, we conclude that CaMKII*δ* likely promotes neuronal survival in response to ischemic stress in the brain. Our findings agree with a previous study showing that CaMKII*δ* expression was elevated in the survival neurons after traumatic brain injury in rat brain, and the increased expression is implicated in the apoptosis of the neuron and the recovery of motor functional outcome.^34^ It has been well documented that chronic loss/inactivation of CaMKII promotes neurotoxicity, whereas acute CaMKII inhibition is neuroprotective.^17, 18, 19, 20, 21, 22, 23, 24^ The mechanisms underlying these opposing effects of CaMKII inhibition on neuronal survival are complex. One hypothesis is that the neuroprotective effect of acute CaMKII inhibition is limited to the excitotoxic challenges as it could paradoxically prevent excitotoxicity-induced CaMKII inactivation-aggregation, as described by Ashpole *et al.*^18^ The diverse functions of CaMKII are also consistent with the dual actions of Ca^2+^ signaling that activate CaMKII in different biological systems.^35^ The I/R-induced upregulation of CaMKII*δ* may act independently or together with other isoforms to alter the function of the CaMKII holoenzyme, thereby affecting the neuronal injury and recovery process after ischemia.

### The CaMKII*δ*/NF-*κ*B signaling pathway in neuronal survival

CaMKII downstream targets may promote excitotoxic cell death or neuronal survival.^12^ We have examined a number of potential downstream pathways, such as NF-*κ*B, Akt, ERK and p38 signaling pathways, which are known to be involved in I/R-induced pathophysiology. Knockdown of *C2dat1* led to the identification of the NF-*κ*B signaling pathway as a primary target of CaMKII*δ* in conferring neuroprotection in N2a cells. I/R-induced activation of the NF-*κ*B signaling pathway was abolished by silencing *C2dat1*, followed by downregulation of CaMKII*δ* and inhibition of IKK*α*/*β* phosphorylation and protein expression. The inhibition of the NF-*κ*B signaling pathway resulted in increased OGD/R-induced cell death, further supporting the pro-survival effect of the NF-*κ*B signaling pathway in N2a cells. The pro-survival effect of NF-*κ*B has been demonstrated in MCAO-based ischemic model by other groups. For example, it has been shown that NF-*κ*B participates in survival signaling following transient focal ischemia (MCAO paradigm) in p502/2 mice showed a clear neuroprotective role in the hippocampus and striatum, in which degenerating neurons were detected 4 days after 1 h of ischemia.^36^ Nonetheless, as a functionally divergent transcription factor, NF-*κ*B has also been shown to contribute to neuronal cell death as part of the pro-inflammatory response, depending on the cerebral ischemia models and experimental approaches.^36, 37, 38^ Schneider *et al.*^38^ reported that NF-*κ*B was activated after 2 h of MCAO followed by 20 h of reperfusion and contributed to I/R-induced neuronal damage. In the heart, CaMKII*δ* has been implicated as a mediator of I/R-induced myocardial injury through the NF-*κ*B pathway.^39^ It is also possible that the NF-*κ*B pathway may exert alternative functions in primary neurons and mouse models of I/R as compared with undifferentiated N2a cells. In addition to the NF-*κ*B pathway, other pro-survival signaling pathways may also be activated by CaMKII*δ*, such as the phosphorylation and activation of nNOS, regulation of several ion channels, activation of ERK and CREB, and inhibition of GSK-3, Bad and HDAC5,^12^ which may have a role in mediating the neuroprotective function of *C2dat1* during ischemia.

In summary, our study reveals a novel lncRNA *C2dat1* that regulates CaMKII*δ* expression in focal cerebral ischemia. *C2dat1* promotes neuronal survival in response to I/R through upregulating CaMKII*δ* expression. The identification of CaMKII*δ* regulatory lncRNAs has shed light to the transcriptional control of CaMKII, one of the most important families of protein kinases for neurons, and may provide new opportunities to study or selectively target a specific CaMKII isoform (CaMKII*δ*) in biological processes.

## Materials and Methods

### Reagents and antibodies

The iScript cDNA synthesis kit and EVA Green SMX 500R were obtained from Bio-Rad Laboratories (Richmond, CA, USA). Cell culture reagents and media were from American Type Culture Collection (ATCC, Rockville, MD, USA). Anti-CaMKII*δ* antibody was purchased from Abcam (Cambridge, MA, USA) and the NF-*κ*B Pathway Sampler Kit and Bcl-xL antibody were purchased from Cell Signaling (Danvers, MA, USA). GAPDH and *α*-tubulin antibodies were from Santa Cruz Biotechnology (Santa Cruz, CA, USA). Goat anti-rabbit and goat anti-mouse HRP-conjugated secondary antibodies were from Promega (Madison, WI, USA).

### Animals

Male C57BL/6J mice (8–10 weeks of age, 18–20 g weight) used for MCAO study were purchased from Guangdong Medical Laboratory Animal Center, Guangzhou, China. The use of animals under this protocol followed all guidelines set forth in the Guangdong Medical Laboratory Animal Center Institutional Animal Use Policy. Mice were housed in cages maintained in a regulated environment (12-h light/dark cycle), supplied with food and water without restriction. Mice used for preparation of primary cortical neuron culture and immunostaining were housed in a temperature-controlled room on a 12-h light/12-h dark cycle with standard mouse diet and water *ad libitum* at the University of Pittsburgh animal facility. The mice at ages 2–3 months were used for fluorescence immunostaining. All studies were in compliance with the guidelines outlined in the Guide for the Care and Use of Laboratory Animals from the US Department of Health and Human Services and were approved by the University of Pittsburgh Medical Center Institutional Animal Care and Use Committee.

### Mouse model of focal cerebral ischemia and reperfusion

Focal cerebral ischemia was induced by intraluminal MCAO using an intraluminal monofilament technique as described previously.^26, 27^ Briefly, mice (18–20 g) were anesthetized with isoflurane (3% initially, 1% to 1.5% maintenance) in O_2_ and N_2_O (1:3) and the rectal temperature was controlled at 37.0±0.5 °C during surgery. After isolating the right common carotid artery (CCA), the right external carotid artery (ECA) and the right internal carotid artery (ICA), a 6-0 suture was tied at the origin of the ECA and at the distal end of the ECA. The left middle cerebral artery (MCA) was occluded by a silicone rubber coated monofilament insertion of a 6-0 nylon monofilament with a thin silicon coat (filament size 6-0, diameter 0.16 mm, length 25 mm; diameter with coating 0.20±0.02 mm; coating length 5 mm), was pushed up the ICA to occlude the origin of the MCA. After 1-h occlusion, it was removed to allow reperfusion for 6, 12 or 24 h with the ECA tied permanently. Sham-operated mice underwent the similar operations to expose the carotid arteries without the suture insertion. After reperfusion, the mice were killed and the brains were removed quickly for biochemical analysis. Some brain tissues were snap frozen in isopentane for cryostat sectioning. Serial coronal sections (1 mm apart) were stained with 2, 3, 5-triphenyltetrazolium chloride monohydrate (TTC; Sigma-Aldrich, St. Louis, MO, USA) for assessing size of infarction. Tissues from the ischemic core and surrounding penumbra were isolated and snap frozen in liquid N_2_ for western blotting.

### Cell culture and OGD/R model

N2a cells were purchased from American Type Culture Collection (ATCC, Manassas, VA, USA) and cultured in Minimum Essential Medium Eagle (MEM) with Eagle's salt and l-glutamine supplemented with 10% FBS (Invitrogen, Carlsbad, CA, USA) at 37 °C with 5% CO_2_. The primary neuron culture was prepared as described previously.^27^ Cortical tissues were obtained from 14 to 16 day-old C57BL/6 mouse embryos.

To mimic ischemic-like conditions *in vitro*, the growth medium was replaced with deoxygenated glucose-free Hanks' Balanced Salt Solution (Invitrogen). Cells were placed into a temperature-controlled (37±1 °C) anaerobic chamber (Forma Scientific, Marietta, OH, USA) that contains a gas mixture composed of 5% CO_2_ and 95% N_2_ for 3 h (N2A)^40^ or 1 h (primary neuronal cells).^27^ After OGD exposure, the medium was replaced with glucose-containing growth medium containing 10% FBS and cultured under normoxic condition for several different time points at 37 °C with 5% CO_2_. Control cell cultures were not deprived of oxygen and glucose, and always placed in normoxygenated glucose-containing DMEM.

### Nuclear and cytoplasmic cell fractions

The nuclear and cytoplasmic fractions were prepared as described previously.^41^ Briefly, N2a cells were cultured in 150-mm dishes for 48 h. After OGD/R treatment, N2a cells were harvested and washed with PBS, and then resuspended in ice-cold hypotonic lysis buffer (HLB) (10 mM Tris, pH 7.5, 10 mM NaCl, 3 mM MgCl_2_, 0.3% NP-40). To prepare cytoplasmic fraction, supernatant after the addition of sodium acetate and ethanol was precipitated at –20 °C overnight. To make nucleoplasmic fraction, pelleted nuclei were washed 3x with ice-cold HLB and resuspended in ice-cold modified Wuarin–Schibler buffer (MWS) (10 mM Tris-HCl, pH 7.0, 4 mM EDTA, 0.3 M NaCl, 1 M urea, 1% NP-40). After 30-min incubation on ice, the samples were spun at 500 × g at 4 °C for 5 min and the supernatant was kept as nucleoplasmic fraction. Pelleted chromatin was washed 3x with ice-cold MWS, and RNA was extracted using Trizol (Invitrogen).

### RNA FISH

RNA FISH probe sets were designed by using software available through Stellaris Probe Designer (http://www.biosearchtech.com/stellarisdesigner/), which were labeled with the reporter dye Quasar 570 and synthesized by Biosearch Technologies (Petaluma, CA, USA). RNA FISH staining was performed according to standard Stellaris protocols (www.biosearchtech.com/stellarisprotocols) from the manufacturer.^42^ Briefly, OGD/R-treated N2a cells were fixed and hybridized with Stellaris RNA FISH probes. After hybridization, the N2a cells were counter-stained by DAPI and mounted with Vectashield Mounting Medium (Vector Laboratories, Burlingame, CA, USA; catalog #H1000) for imaging under confocal microscope.

### RNAi transfection

N2a cells were seeded into six-well plates at a density of 3 × 10^4^ cells per well. The siRNAs at 50–75 nM final concentration were transfected into cells using DharmaFECT transfection reagent according to the manufacturer's instructions for 48 h. The lncRNA *C2dat1*-targeted siRNA, *CAMK2D-targeted* siRNA and a control non-targeting siRNA (negative control) were obtained from GE Dharmacon (Lafayette, CO, USA). Two days after transfection, N2a cells were subjected to either control or OGD treatment, followed by reoxygenation under normoxic culture conditions for various times (0, 12 and 24 h).

### Preparation of samples and RNA extraction

For N2a cells, total RNA was extracted using TRIzol LS Reagent according to the manufacturer's instructions. For tissue RNA extraction, the ischemic penumbra and ischemic cortex were taken as experimental samples as described previously.^43^ Briefly, the brain cerebral cortex was sectioned into three slices, section 1 is ischemic cortex; section 2 is ischemic penumbra and section 3 is the rest part of brain tissue. The section 1 and section 2 were used for RNA extraction, respectively. This was done to avoid mesial hemispheric structures, where the blood supplied primarily by the anterior cerebral artery. RNA extraction was carried out with TRIzol LS Reagent according to the manufacturer's instructions. RNA quantity and quality were measured by Synergy H1 Hybrid Reader (BioTek, Winooski, VT, USA). The value of OD260/280 is around 1.8 as a criterion of acceptable purity and RNA integrity was assessed using standard denaturing agarose gel electrophoresis. The total amount of RNA obtained did not differ significantly between the samples.

### Immunostaining and binary image analysis

Fluorescence immunostaining on brain tissue sections was conducted as described previously.^27^ Briefly, mouse tissue sections were incubated with blocking solution containing either rabbit anti-CaMKII*δ* (1 : 100) or mouse anti-MAP-2 (1 : 100). Sections were washed with TBS for 30 min and then incubated with the following secondary antibodies (1 : 200): goat anti-rabbit Alexa Fluor 488-conjugated IgG for CaMKII*δ* (1 : 200) and goat anti-mouse Alexa Fluor 546 conjugated IgG for MAP-2 (1 : 100). The nuclei were stained with ToPro-3 (1 : 1000 in blocking solution) and mounted with Vectashield (Vector Laboratories). Brain sections stained with secondary antibody only were used as negative controls. The imaging method and the binary image analysis were described previously.^27^

### Primers

A list of real-time RT-PCR primers used in this study and their respective sequences were described in the Supplementary Table S1.

### Real-time RT-qPCR

One microgram of total RNAs was used to generate cDNA using the iScript cDNA synthesis kit. Real-time PCR was subsequently performed using the SsoFast EvaGreen Supermix on CFX96 Real-Time PCR Detection System (Bio-Rad, Richmond, CA, USA) to analyze the expression of *C2dat1* and *CAMK2D* transcripts using the primers listed in Supplementary Table S1. Data were normalized using GAPDH as control.

### Western blot analysis

Cells were harvested and lysed in lysis buffer (50 mM Tris-HCl, pH 7.4, 150 mM NaCl, 1.5 mM MgCl_2_, 10% glycerol, 1% Triton X-100, 5 mM EGTA, 20 *μ*M leupeptin, 1 mM AEBSF, 1 mM NaVO_3_, 10 mM NaF and 1 × protein inhibitor cocktail). Western blot analysis was carried out as previously reported.^44^ Briefly, 20 *μ*g of proteins were separated on SDS-PAGE and transferred onto nitrocellulose membranes. After blocking, the membranes were incubated with the appropriate primary antibody at 4 °C overnight. After washing, the membranes were incubated with secondary antibody at room temperature for 1 h. Proteins were detected with the enhanced chemiluminescence (ECL) kit. The images were captured on X-ray film and the band intensity was analyzed by Image J software (NIH, Bethesda, MD, USA).

### Cell survival assay

Cell survival was assayed by Cell Counting Kit-8 (Dojindo Laboratories, Kumamoto, Japan), according to the manufacturer's instructions. N2a cells were plated at a density of 1 × 10^5^ cells per well in 24-well plates. After siRNA transfection and/or drug treatment, CCK-8 solution containing a highly water-soluble tetrazolium salt WST-8 [2-(2-methoxy-4-nitrophenyl)-3-(4-nitrophenyl)-5-(2,4-disulfophenyl)-*2H*-tetrazolium, monosodium salt] was added to cells in each well, followed by incubation for 1–4 h. Cell proliferation/viability was determined by measuring the OD at 450 nm. Percent over control was calculated as a measure of cell viability.

### Statistical analysis

All statistical analysis was done using GraphPad Prism IV software (GraphPad Software, La Jolla, CA, USA). A *P*-value <0.05 was considered statistically significant (*P<0.05; **P<0.01; ***P<0.001; ****P<0.0001; ns, not significant).

## Abbreviations

LncRNA: long non-coding RNA
I/R: ischemia/reperfusion
*C2dat1*: *CAMK2D*-associated transcript 1
OGD/R: oxygen-glucose deprivation/reoxygenation
tPA: tissue plasminogen activator
ncRNA: non-coding RNA
miRNAs: microRNAs
CNS: central nervous system
CaMKII: calcium/calmodulin-dependent kinase II
MCAO: middle cerebral artery occlusion
Map2: microtubule-associated protein-2
FISH: fluorescence *in situ* hybridization
si-*C2dat1*: *C2dat1*-targeting siRNA
CCA: common carotid artery
ECA: external carotid artery
ICA: internal carotid artery
MCA: middle cerebral artery
HLB: hypotonic lysis buffer
MWS: modified Wuarin–Schibler buffer
ECL: enhanced chemiluminescence
si-NT: non-targeting siRNA
si-*CAMK2D*: *CAMK2D*-targeting siRNA
