# Supplementary figures and images for: Long non-coding RNA C2dat1 regulates CaMKIIδ expression to promote neuronal survival through the NF-κB signaling pathway following cerebral ischemia
Source: Cell Death Dis. 2016 Mar 31;7(3):e2173–. doi: 10.1038/cddis.2016.57 (PMC4823958; doi:10.1038/cddis.2016.57)

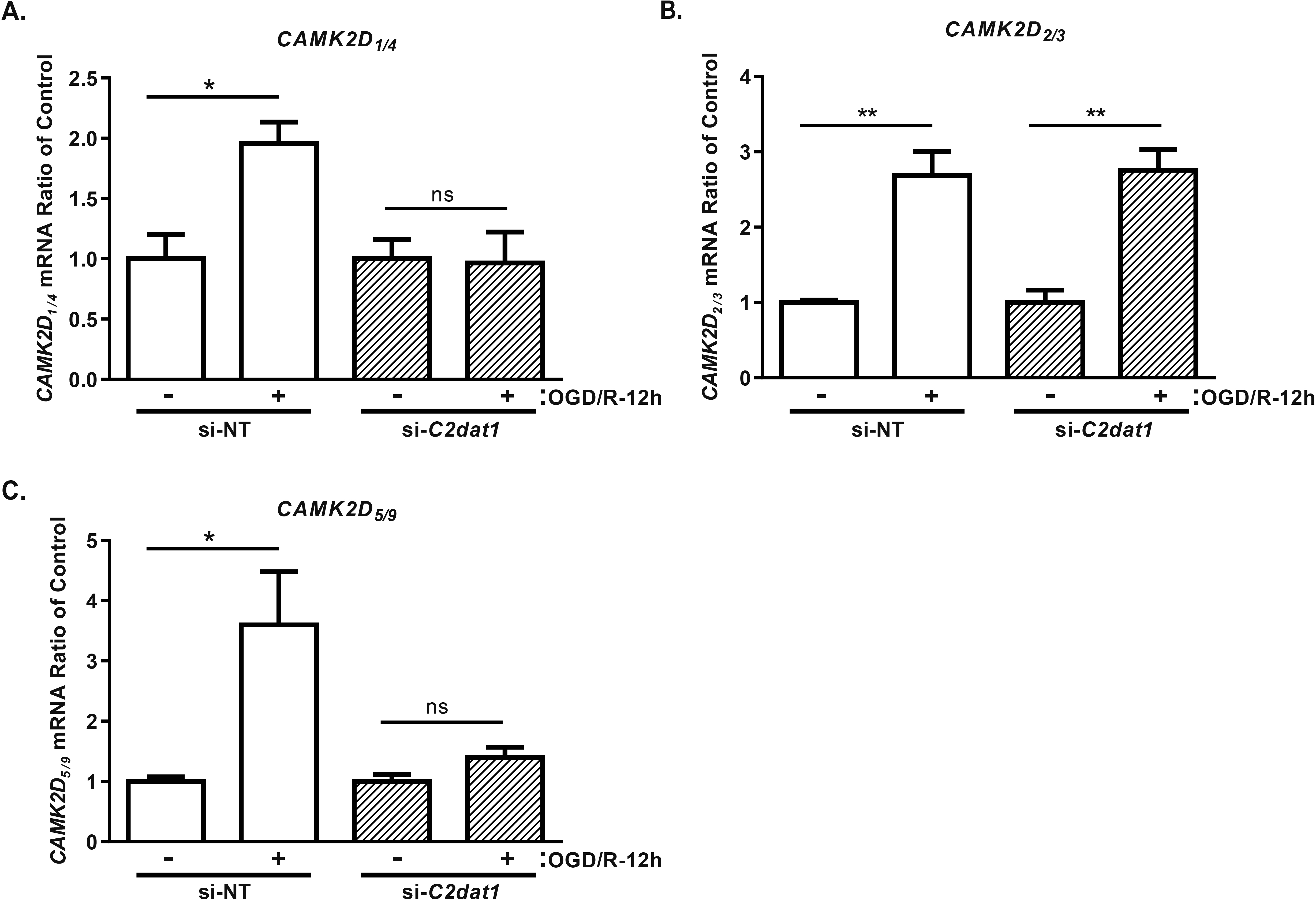

Supplement: Supplementary Figure 1 [file cddis201657x1.tif]

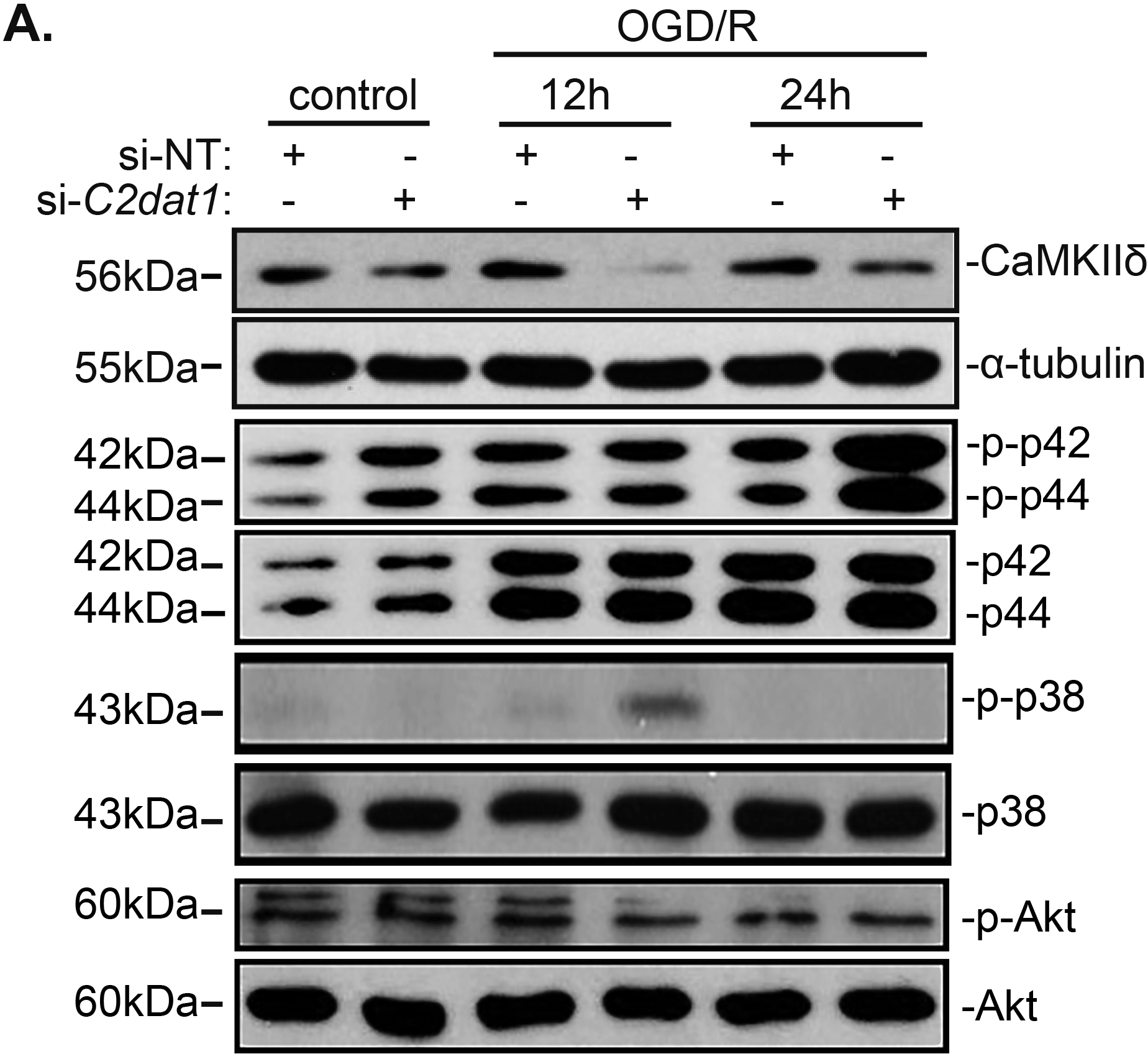

Supplement: Supplementary Figure 2 [file cddis201657x2.tif]

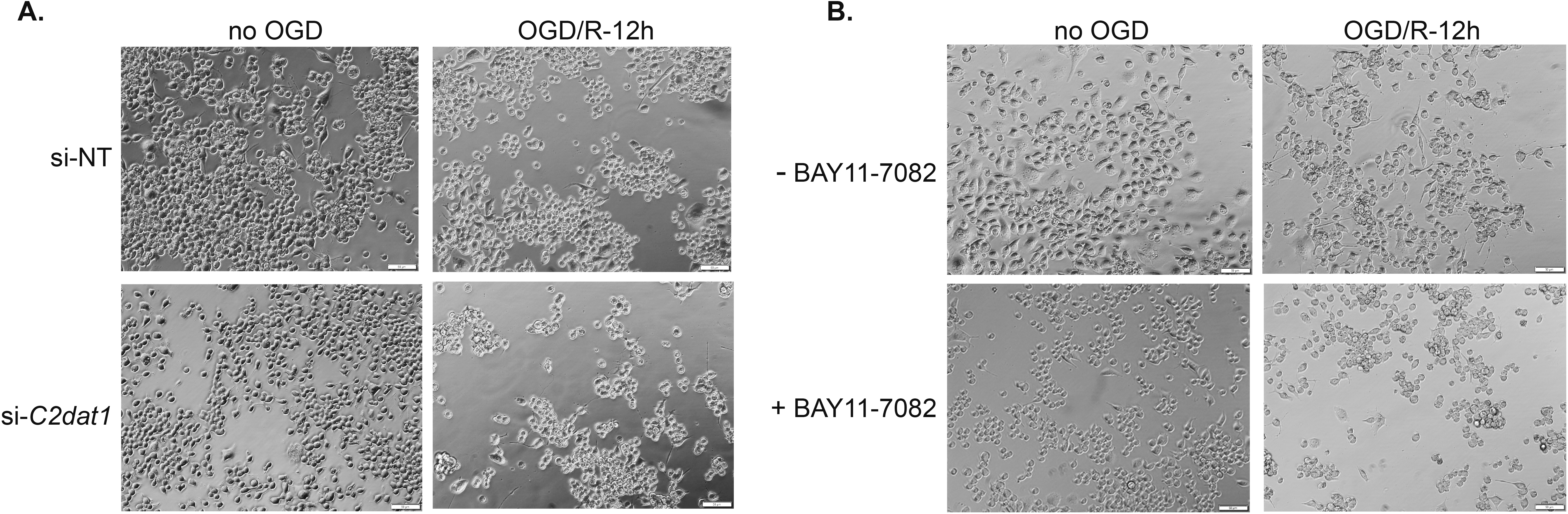

Supplement: Supplementary Figure 3 [file cddis201657x3.tif]
