# Supplementary material for: Long non-coding RNA C2dat1 regulates CaMKIIδ expression to promote neuronal survival through the NF-κB signaling pathway following cerebral ischemia
Source: Cell Death Dis. 2016 Mar 31;7(3):e2173–. doi: 10.1038/cddis.2016.57 (PMC4823958; doi:10.1038/cddis.2016.57)
Supplement: Supplementary Table 1 [file cddis201657x4.docx]

**Table S1. List of real time PCR primers used in the study.**

| Mouse lncRNA or mRNA | Forward primer (5′–3′) | Reverse primer (5′–3′) | product size |
| --- | --- | --- | --- |
| AK134201 | TTCGGCCAGGTTTCTCCAAG | CTTGCTACCTCCTTCCCTGC | 110 |
| AK131672 | GGACTGTGGGTAGTGTGCTC | TACCGCTAGTCTGGCTTGTG | 154 |
| AK153573 | ATGTAAGGGCTTGGTCCCAC | TGTAGCCTCCGTGTGAAAGG | 116 |
| AK135044 | TGCAGTTGGAGTTGCTTGGA | AGGAAACGACAGCAGAAGGG | 198 |
| AK048215 | CCCGGTTCCTCTTCCAGAAC | CAATGATCGGGGTCCTGGTG | 127 |
| AK051903 | ATGAACTGCTCCTGGTGACG | TGCATGGCTGTTTAGCCAGA | 160 |
| AK013548 | CAAGAAGCGCAAAGTGTCCC | CCTCTTGCCCCTTCGTCTAC | 124 |
| AK161159 | GTCCAAAATGGTGGGGCAAC | GGTCGGGTAGAATCCCTGGT | 98 |
| AK084575 | CTGGGAAGCAAGGAGGTCTG | TGAGGGGCCAAGTGTTTCTG | 143 |
| AK040843 | CGCACACTCCAGTGAAGACA | TTGGAGAACCTACCGCCTCT | 109 |
| RAD23a | AATTAGCCGTCACCAGGAGC | GCGGTGTCACCTGGATATAGT | 140 |
| CAMK2D | TATCGGAGGAGGGCTTCCAT | ACTGGCATCAGCTTCACTGT | 104 |
| GAPDH | GGACCTCATGGCCTACATGG | TAGGGCCTCTCTTGCTCAGT | 85 |
