# Supplementary material for: Long non-coding RNA C2dat1 regulates CaMKIIδ expression to promote neuronal survival through the NF-κB signaling pathway following cerebral ischemia
Source: Cell Death Dis. 2016 Mar 31;7(3):e2173–. doi: 10.1038/cddis.2016.57 (PMC4823958; doi:10.1038/cddis.2016.57)
Supplement: Supplementary Table 2 [file cddis201657x5.docx]

**Table S2. Selected rat LncRNAs and their corresponding mouse homolog.** A list of selected differentially expressed rat lncRNAs identified on the rat lncRNA array are shown along with their mouse homolog and associated gene and protein names. These lncRNAs were further validated using the Neuro-2a cells.

| **Rat LncRNA gene name** | **Rat Associated gene acc#** | **Rat Associated gene name** | **FC (abs)** | **Regulation** | **P-value** | **Mouse lncRNA gene name** | **Mouse associated mRNA acc#** | **Mouse associated protein name** |
| --- | --- | --- | --- | --- | --- | --- | --- | --- |
| AK040843 | NM_031327 | Cyr61 | 7.396903 | up | 0.0017541 | AK040843 | [NM_010516.2](http://www.ncbi.nlm.nih.gov/nucleotide/239937453?report=genbank&log$=nucltop&blast_rank=1&RID=8HSSXGGE015) | Cyr61 |
| AK134201 | NM_001107084 | Bcl6 | 6.01786 | up | 0.0071732 | AK134201 | [NM_009744.3](http://www.ncbi.nlm.nih.gov/nucleotide/142360700?report=genbank&log$=nucltop&blast_rank=1&RID=8HAH3NXV01R) | Bcl6 |
| AK153573 | NM_012519 | Camk2d | 3.6980176 | up | 0.0166668 | AK153573 | [NM_001025439.1](http://www.ncbi.nlm.nih.gov/nucleotide/70906478?report=genbank&log$=nucltop&blast_rank=5&RID=8HRCTHHC01R) | Camk2d |
| AK135044 | NM_053343 | Dclk1 | 3.0228415 | up | 0.0169237 | AK135044 | [NM_001111051.1](http://www.ncbi.nlm.nih.gov/nucleotide/161353458?report=genbank&log$=nucltop&blast_rank=1&RID=8HPT0HJ201R) | Dclk1 |
| AK048215 | NM_001013989 | G3bp2 | 12.376367 | down | 3.07E-04 | AK048215 | [NM_001080795.1](http://www.ncbi.nlm.nih.gov/nucleotide/124248569?report=genbank&log$=nucltop&blast_rank=1&RID=8HPHUY3M014) | G3bp2 |
| AK051903 | NM_001013190 | Rad23a | 2.8896892 | down | 0.00640559 | AK051903 | [NM_009010.4](http://www.ncbi.nlm.nih.gov/nucleotide/145966817?report=genbank&log$=nucltop&blast_rank=1&RID=8HPFFD1T01R) | Rad23a |
| AK161159 | NM_032062 | Kalrn | 3.4084017 | down | 0.010159032 | AK161159 | [NM_177357.3](http://www.ncbi.nlm.nih.gov/nucleotide/295054243?report=genbank&log$=nucltop&blast_rank=1&RID=8HP3BBB701R) | Kalrn |
| AK084575 | NM_024357 | Htt | 2.9364972 | down | 0.008170723 | AK084575 | [NM_010414.2](http://www.ncbi.nlm.nih.gov/nucleotide/315221149?report=genbank&log$=nucltop&blast_rank=1&RID=8HNX4RGE01R) | Huntingtin |
