# Supplementary material for: Long non-coding RNA C2dat1 regulates CaMKIIδ expression to promote neuronal survival through the NF-κB signaling pathway following cerebral ischemia
Source: Cell Death Dis. 2016 Mar 31;7(3):e2173–. doi: 10.1038/cddis.2016.57 (PMC4823958; doi:10.1038/cddis.2016.57)
Supplement: Supplementary Table 3 [file cddis201657x6.docx]

| **Antibody name** | **MW (kDa)** | **Vendor** | **Cat#** | **Dilution** |
| --- | --- | --- | --- | --- |
| Bcl-xl(54H6) Rabbit mAb | 30 | Cell Signaling | #2764 | 1:1000 |
| Phospho-IKKα/β(Ser176/180)(16A6) Rabbit mAb | 85,87 | Cell signaling | #2697 | 1:1000 |
| IKKα antibody | 85 | Cell signaling | #2682 | 1:1000 |
| IKKβ antibody | 87 | Cell signaling | #2678 | 1:1000 |
| IκBα (L35A5) Mouse mAb antibody | 39 | Cell signaling | #4814 | 1:1000 |
| Phospho- IκBα (Ser32)(14D4) Rabbit mAb | 40 | Cell signaling | #2859 | 1:1000 |
| NF-κB p65(D14E12)XP Rabbit mAb | 65 | Cell signaling | #8242 | 1:1000 |
| Phospho-p38 MARK (Thr180/Tyr182)(D3F9)XP Rabbit mAb | 43 | Cell signaling | #4511 | 1:1000 |
| Phospho-Akt(Ser473) antibody | 60 | Cell signaling | #9271 | 1:1000 |
| p38 MARK(Thr180/Tyr182)(D3F9)XP Rabbit mAb | 43 | Cell signaling | #9212s | 1:1000 |
| Akt antibody | 60 | Cell signaling | #9272 | 1:1000 |
| Phospho-p44/42MARK(Erk1/2)(Thr202/Tyr204) antibody | 42,44 | Cell signaling | #9101 | 1:1000 |
| p44/42MARK(Erk1/2) antibody | 42,44 | Cell signaling | #9102 | 1:1000 |
| GAPDH antibody | 36 | Enzo | ADI-CSA-335-E | 1:3000 |
| α-tubulin | 55 | Santa Cruz | sc-8035 | 1:3000 |
| Anti-CaMKIIδ antibody | 56 | Abcam | Ab105502 | 1:2000 |
| Anti-rabbit IgG Antibody | All | Cell signaling | #7074 | 1:1000-1:3000 |
| Anti-mouse IgG Antibody | All | Cell signaling | #7076 | 1:1000-1:3000 |

**Table S3. List of all antibodies used in the study.**
