# Supplementary material for: Long non-coding RNA C2dat1 regulates CaMKIIδ expression to promote neuronal survival through the NF-κB signaling pathway following cerebral ischemia
Source: Cell Death Dis. 2016 Mar 31;7(3):e2173–. doi: 10.1038/cddis.2016.57 (PMC4823958; doi:10.1038/cddis.2016.57)
Supplement: Supplementary Information [file cddis201657x7.doc]

**Supplemental Information**

**Table S1.** List of real time PCR primers used in the study.

**Table S2.** Selected rat LncRNAs and their corresponding mouse homolog.

**Table S3.** List all the antibodies used in the study, including vendor, cat# and dilution.

**Figure S1.** **Effects of *C2dat1* knockdown on the expression of multiple *CAMK2D* isotypes in N2a cells.** The cells were transfected with *C2dat1*-targeting siRNA (si-*C2dat1*) or a non-targeting siRNA (si-NT). Two days after the transfection, cells were subjected to OGD/R for 12 h. The transcript levels of *CAMK2D*_1/4_ (**A**), *CAMK2D*_2/3_ (**B**), and *CAMK2D*_5/9_ (**C**) were examined by real-time RT-qPCR. The data are the Mean±SEM of representative experiment performed in triplicate.*, *P*<0.05; **, *P*<0.01; ns, not significant.

**Figure S2. Effects of *C2dat1* knockdown on the expression and activity of Akt, ERK, and p38 signaling pathways.** N2a cells were transfected with a si-*C2dat1* and si-NT. The transfected cells were subjected to OGD/R, followed by immunoblotting for native and phospho-Akt, -ERK, and -p38. Representative images from one of at least three independent experiments are shown.

**Figure S3. A.** Knockdown of *C2dat1* promoted OGD/R-induced cell death. Representative images of cells in Fig. 6A before and after OGD/R. **B.** Inhibition of IKK by BAY11-7082 enhanced cell death induced by OGD/R in N2a cells. Representative images in Fig. 6B before and after BAY11-7082 treatment are shown.
